# Supplementary figures and images for: Differences between Obese and Non-Obese Children and Adolescents Regarding Their Oral Status and Blood Markers of Kidney Diseases
Source: J Clin Med. 2021 Aug 21;10(16):3723. doi: 10.3390/jcm10163723 (PMC8397190; doi:10.3390/jcm10163723)

Figure 1. Flow chart of the study.

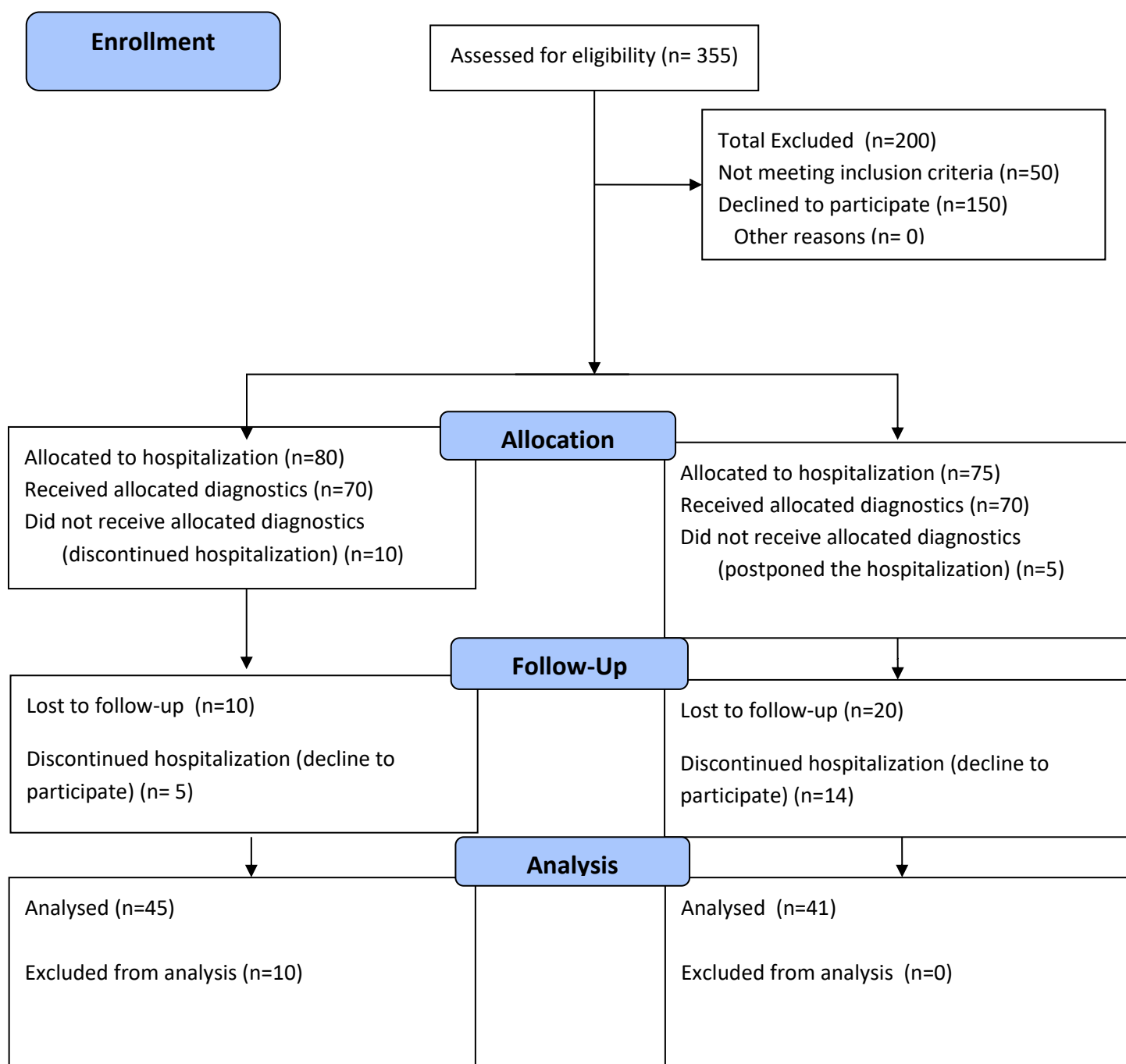

Supplement: Supplementary file 1 [file jcm-10-03723-s001.zip › supplementary figures/figure S1.pdf]
